# Burnout and its associated factors in healthcare workers during the COVID-19 pandemic

**DOI:** 10.1192/j.eurpsy.2023.1702

**Published:** 2023-07-19

**Authors:** N. Halouani, M. Koukane, O. Elleuch, M. Turki, A. Samet, F. Jemil, S. Ellouze, J. Aloulou

**Affiliations:** Psychiatry B, Hedi Chaker University Hospital of Sfax, Sfax, Tunisia

## Abstract

**Introduction:**

Due to the COVID-19 pandemic, our health system had to face new challenges such as Burnout (BO), particularly among healthcare workers (HCWs).

**Objectives:**

Our study aimed to examine the prevalence of burnout among HCWs, as well as to identify the sociodemographic and professional factors associated to it.

**Methods:**

This was a cross-sectional descriptive and analytical survey, conducted during the fifth wave of COVID-19 between December 2021 and February 2022, among the healthcare workers of 3 hospitals in Djerba city. We used an online questionnaire to collect their sociodemographic and professional data. Maslach Burnout Inventory (MBI) was used to assess their burnout level.

**Results:**

Our population consisted of 95 HCWs with a sex ratio of 0.46. Most of them (75%) had an age of less than 40 years. Among our participants, 56.8% were paramedics and 71.6% had a working experience of less than 10 years. A rate of 56.8% worked full-time with a minimum of 36 hours per week and more than 5 on-calls per month in 56.8%.

In our study, 76% of the HCWs were affected by BO. The mean emotional exhaustion, depersonalization and professional accomplishment scores were 35.74 ±12.16, 11.84 ±8.08 and 27.32 ±6.95, respectively. A rate of 69% had a high level of emotional exhaustion, 42% had a high level of depersonalization and 41% had a low level of personal accomplishment.

The subgroup analysis showed that BO was associated with: an age of less than 30 years (p=0.007); having no children (p=0.030); a work experience of less than 10 years (p=0.001); a number of working hours greater than 36 hours (p=0.030) and a number of on-call duties greater than 5 per month (p=0.007).

**Conclusions:**

Our study shows the burden of BO in the HCWs of Djerba, especially among the youngest ones, the least experienced and those with the highest workload. Thus, a special attention must be paid to this condition, and the implementation of a prevention strategy is essential.

**Disclosure of Interest:**

None Declared

